# Supplementary material for: Comparing the performance of a large language model and naive human interviewers in interviewing children about a witnessed mock-event
Source: PLoS One. 2025 Feb 28;20(2):e0316317. doi: 10.1371/journal.pone.0316317 (PMC11870376; doi:10.1371/journal.pone.0316317)
Supplement: S1 Table — (DOCX) [file pone.0316317.s001.docx]

**S1 Table**

**Content of the Mock-Event Videos.**

| Number | Gender of Child | Adult taking photo of the child | Physical contact |
| --- | --- | --- | --- |
| 1 | Male | × | √ |
| 2 | Male | × | × |
| 3 | Male | √ | × |
| 4 | Female | × | √ |
| 5 | Female | × | × |
| 6 | Female | √ | × |
