# Supplementary material for: Comparing the performance of a large language model and naive human interviewers in interviewing children about a witnessed mock-event
Source: PLoS One. 2025 Feb 28;20(2):e0316317. doi: 10.1371/journal.pone.0316317 (PMC11870376; doi:10.1371/journal.pone.0316317)
Supplement: S2 Table — (DOCX) [file pone.0316317.s002.docx]

**S2 Table**

**Coding of Static and Dynamic Elements in Child Answers**

**Coding of Static Elements in Child Answers**

| **Category** | **Detail** | **Outcome** |
| --- | --- | --- |
| Right | Not Mentioned | One Correct |
| Right | Right | Two Correct |
| Right | False | One Incorrect |
| False | / | One Confabulation |

**Coding of Dynamic Elements of Characters in Child Answers**

| **Subject** | **Action** | **Tool** | **Object** | **Content** |
| --- | --- | --- | --- | --- |
| Adult | Ask | / | Child | “What animals do you like most?” |
| Adult | Take a picture | Camera | Cup | / |
| Child | Answer | / | Adult | “I like panda most.” |
| …… | …… | …… | …… | …… |
