# Supplementary material for: Comparing the performance of a large language model and naive human interviewers in interviewing children about a witnessed mock-event
Source: PLoS One. 2025 Feb 28;20(2):e0316317. doi: 10.1371/journal.pone.0316317 (PMC11870376; doi:10.1371/journal.pone.0316317)
Supplement: S3 Table — (DOCX) [file pone.0316317.s003.docx]

**S3 Table**

**Interview Transcripts for the Most Typical LLMs and Human Interviewer Interviews.**

| **Type** | **Number** | **Question** | **Category^a^** | **Answer** | **Correct** | **FALSE** |
| --- | --- | --- | --- | --- | --- | --- |
| LLM | 1 | Can you tell me what you see in the video? | 5 | I saw it, he picked the cell phone, and took a picture of the fan. And he likes snowball fights in the winter. | 4 | 0 |
|  | 2 | How did the children and adults in the video choose their cell phones? Did they say anything? | 1 | They said I'd pick any one of these three devices, and picked the cell phone. | 4 | 0 |
|  | 3 | How did they start playing the game after they chose the phone? | 3 | Turned on the camera, then took a picture of an electric fan, and pointed it at the camera. | 3 | 0 |
|  | 4 | Did you notice anything special about the adult or the clothes they were wearing in the video? | 1 | Adults wore hats and also glasses, and children wore short sleeves. | 3 | 0 |
|  | 5 | About the adult, what did his hat and glasses look like? Are there any special features, such as a beard, hairstyle, or the color of the hat? | 1 | The color seems to be…I don’t know. | 0 | 0 |
|  | 6 | What do the adult's eyeglasses look like? Are they regular glasses or sunglasses? | 4 | Regular glasses. | 1 | 0 |
|  | 7 | Are there any other interactions or conversations in the video besides the part where the fan is shot? | 3 | Just asked which season you like and then the kid said I like winter because I can have snowball fights. | 6 | 0 |
|  | 8 | Were there any other special episodes or anything in the video that caught your attention? | 3 | There's no more. | 0 | 0 |
|  | 9 | Do you think these questions I just asked you here, or are they computer generated? | / | I think it was you who asked. | 0 | 0 |
| Human Interviewer | 1 | What do you see in this video? How do you feel after seeing this video? | 3 | Nothing feels. | 0 | 0 |
|  | 2 | Have you experienced what happened in this video? | 4 | Nothing. | 0 | 0 |
|  | 3 | Can you tell me what kind of video you saw? | 3 | I saw it. She played a game with her. | 3 | 0 |
|  | 4 | After she played a game, did she feel good? | 4 | Good. | 0 | 1 |
|  | 5 | What kind of game did they play? | 3 | They picked something, took a picture of it, and then showed a picture of it. | 7 | 0 |
|  | 6 | Can you be more specific? | 5 | They had three devices and then she chose and then went to take a picture. The shot was of a fan, and then just gave a shot of her video. | 9 | 0 |
|  | 7 | Did she participate in this game with an older friend? | 4 | Right. | 1 | 0 |
|  | 8 | Was the adult very friendly and motivated the child to play? | 4 | Humm… | 0 | 1 |
|  | 9 | Is it not very friendly? Is it friendly or unfriendly? | 4 | Friendly. | 0 | 1 |
|  | 10 | What kind of person do you think the big friend in the video is? | 3 | Very funny guy. | 0 | 1 |
|  | 11 | If it were you, would you choose to play the game with such a big friend? | 4 | No. | 0 | 1 |
|  | 12 | Oh, why? | 3 | Because I have a lot of other people I hang out with. | 0 | 1 |
| *Note:* The number of questions, proportion of recommended questions, proportion of unique information, and number of false information (the main variables with significant differences) were z-standardized within group and the mean standardized score was calculated. The interview with the mean z-score closest to 0 in both groups was selected.  ^a^0: Unrelated to the scenarios in the video, 1: Posing two questions at a time, 2: Repeated question, 3: Question includes indicative words, 4: Close-ended question, 5: Open-ended question | | | | | | |
