# Supplementary material for: Comparing the performance of a large language model and naive human interviewers in interviewing children about a witnessed mock-event
Source: PLoS One. 2025 Feb 28;20(2):e0316317. doi: 10.1371/journal.pone.0316317 (PMC11870376; doi:10.1371/journal.pone.0316317)
Supplement: Attachment — Submitted filename: ReviewerComments - PONE - LLM & Child Interview.docx [file pone.0316317.s004.docx]

Dear Editor and Reviewers:

Thank you for your thoughtful and constructive feedback on our manuscript. We have carefully considered all suggestions and made appropriate revisions to address each point raised. In the following section, we provide detailed responses to your comments, with each response preceded by "Response." The corresponding modifications in the manuscript are highlighted in blue to facilitate review. Both a track-changes version and a clean version of the revised manuscript have been uploaded to the system.

All best,
Authors

Journal Requirements:

**When submitting your revision, we need you to address these additional requirements.**

**1. Please ensure that your manuscript meets PLOS ONE's style requirements, including those for file naming. The PLOS ONE style templates can be found at https://journals.plos.org/plosone/s/file?id=wjVg/PLOSOne_formatting_sample_main_body.pdf and https://journals.plos.org/plosone/s/file?id=ba62/PLOSOne_formatting_sample_title_authors_affiliations.pdf**

Response: Thank you for your suggestion, we have now followed the formatting requirements of the journal and adjusted the basic format of the article

**2. We note that the grant information you provided in the ‘Funding Information’ and ‘Financial Disclosure’ sections do not match. When you resubmit, please ensure that you provide the correct grant numbers for the awards you received for your study in the ‘Funding Information’ section.**

Response: Thank you for your advice. The information we uploaded in the system during our initial submission was incorrect, and we have now corrected the corresponding information in the funding information section of the system

**3. Thank you for stating the following financial disclosure: “This work was supported by the Sundell Foundation and the Shanghai Frontiers Science Center of Artificial Intelligence and Deep Learning at NYU Shanghai.” Please state what role the funders took in the study. If the funders had no role, please state: "The funders had no role in study design, data collection and analysis, decision to publish, or preparation of the manuscript." If this statement is not correct you must amend it as needed. Please include this amended Role of Funder statement in your cover letter; we will change the online submission form on your behalf.**

Response: “The funders had no role in study design, data collection and analysis, decision to publish, or preparation of the manuscript.” We now also added this statement in the cover letter.

**4. Please note that your Data Availability Statement is currently missing the repository name and/or the DOI/accession number of each dataset OR a direct link to access each database. If your manuscript is accepted for publication, you will be asked to provide these details on a very short timeline. We therefore suggest that you provide this information now, though we will not hold up the peer review process if you are unable.**

Response: Thanks to your suggestion, we have now created an anonymous view-only link to the OSF platform to make it easier for you or other researchers to access the study data through this public link.

https://osf.io/5whc7/?view_only=9377fd48c46a412e95a23d08b1e846c3

**5. When completing the data availability statement of the submission form, you indicated that you will make your data available on acceptance. We strongly recommend all authors decide on a data sharing plan before acceptance, as the process can be lengthy and hold up publication timelines. Please note that, though access restrictions are acceptable now, your entire data will need to be made freely accessible if your manuscript is accepted for publication. This policy applies to all data except where public deposition would breach compliance with the protocol approved by your research ethics board. If you are unable to adhere to our open data policy, please kindly revise your statement to explain your reasoning and we will seek the editor's input on an exemption. Please be assured that, once you have provided your new statement, the assessment of your exemption will not hold up the peer review process.**

Response: Thanks to your suggestion, we have decided to make the data from this study publicly available now. You can view the data from this study at the link below:

https://osf.io/5whc7/?view_only=9377fd48c46a412e95a23d08b1e846c3

**6. Please include your full ethics statement in the ‘Methods’ section of your manuscript file. In your statement, please include the full name of the IRB or ethics committee who approved or waived your study, as well as whether or not you obtained informed written or verbal consent. If consent was waived for your study, please include this information in your statement as well.**

Thanks to your suggestion, we have included the full name of the IRB that passed this study in the methods section: New York University (Shanghai)'s Institutional Review Board.

**7. Please remove your figures from within your manuscript file, leaving only the individual TIFF/EPS image files, uploaded separately. These will be automatically included in the reviewers’ PDF.**

Thank you for your advice. We are now removing the figure from the article and uploading it as a separate figure file.

**8. Please include captions for your Supporting Information files at the end of your manuscript, and update any in-text citations to match accordingly. Please see our Supporting Information guidelines for more information: http://journals.plos.org/plosone/s/supporting-information.**

**9. We note that this data set consists of interview transcripts. Can you please confirm that all participants gave consent for interview transcript to be published? If they DID provide consent for these transcripts to be published, please also confirm that the transcripts do not contain any potentially identifying information (or let us know if the participants consented to having their personal details published and made publicly available). We consider the following details to be identifying information: - Names, nicknames, and initials - Age more specific than round numbers - GPS coordinates, physical addresses, IP addresses, email addresses - Information in small sample sizes (e.g. 40 students from X class in X year at X university) - Specific dates (e.g. visit dates, interview dates) - ID numbers Or, if the participants DID NOT provide consent for these transcripts to be published: - Provide a de-identified version of the data or excerpts of interview responses - Provide information regarding how these transcripts can be accessed by researchers who meet the criteria for access to confidential data, including: a) the grounds for restriction b) the name of the ethics committee, Institutional Review Board, or third-party organization that is imposing sharing restrictions on the data c) a non-author, institutional point of contact that is able to field data access queries, in the interest of maintaining long-term data accessibility. d) Any relevant data set names, URLs, DOIs, etc. that an independent researcher would need in order to request your minimal data set. For further information on sharing data that contains sensitive participant information, please see: https://journals.plos.org/plosone/s/data-availability#loc-human-research-participant-data-and-other-sensitive-data If there are ethical, legal, or third-party restrictions upon your dataset, you must provide all of the following details (https://journals.plos.org/plosone/s/data-availability#loc-acceptable-data-access-restrictions): 1. A complete description of the dataset 2. The nature of the restrictions upon the data (ethical, legal, or owned by a third party) and the reasoning behind them 3. The full name of the body imposing the restrictions upon your dataset (ethics committee, institution, data access committee, etc) 4. If the data are owned by a third party, confirmation of whether the authors received any special privileges in accessing the data that other researchers would not have 5. Direct, non-author contact information (preferably email) for the body imposing the restrictions upon the data, to which data access requests can be sent**

Response: Thank you for your advice. We have determined that the data that is now publicly available does not contain any personally identifiable information described above, and the data disclosure plan has been noted in the IRB application, so the current data can be made directly available to the public.

[Note: HTML markup is below. Please do not edit.]

Reviewers' comments:

Reviewer's Responses to Questions

**Comments to the Author**

1. Is the manuscript technically sound, and do the data support the conclusions?

The manuscript must describe a technically sound piece of scientific research with data that supports the conclusions. Experiments must have been conducted rigorously, with appropriate controls, replication, and sample sizes. The conclusions must be drawn appropriately based on the data presented.

Reviewer #1: Partly

Reviewer #2: Partly

2. Has the statistical analysis been performed appropriately and rigorously?

Reviewer #1: No

Reviewer #2: I Don't Know

3. Have the authors made all data underlying the findings in their manuscript fully available?

The [PLOS Data policy](http://www.plosone.org/static/policies.action#sharing) requires authors to make all data underlying the findings described in their manuscript fully available without restriction, with rare exception (please refer to the Data Availability Statement in the manuscript PDF file). The data should be provided as part of the manuscript or its supporting information, or deposited to a public repository. For example, in addition to summary statistics, the data points behind means, medians and variance measures should be available. If there are restrictions on publicly sharing data—e.g. participant privacy or use of data from a third party—those must be specified.

Reviewer #1: Yes

Reviewer #2: Yes

4. Is the manuscript presented in an intelligible fashion and written in standard English?

PLOS ONE does not copyedit accepted manuscripts, so the language in submitted articles must be clear, correct, and unambiguous. Any typographical or grammatical errors should be corrected at revision, so please note any specific errors here.

Reviewer #1: Yes

Reviewer #2: Yes

5. Review Comments to the Author

**Reviewer #1:**

**I am in favor of the publication of this manuscript in PLOS ONE. However, I request modifications to enhance the impact and relevance of this study. This will encourage further research in this field and lead the authors and other teams to continue this (very relevant) work. The subject is relevant, interesting, and well-suited to our contemporary times. The involvement of AI in conducting interviews is a fundamental aspect that is neglected and very rarely explored by scientists. The questions raised by this team of researchers are intriguing and lead to new questions that, I hope, will find answers soon. In other words, this article represents a source of inspiration and motivation for contemporary research in forensic psychology applied to interview methods.**

Response: Thank you for your encouraging review. We appreciate your recognition of this work's potential impact on forensic psychology research. Your emphasis on the nascent nature of AI-assisted interviewing and its importance in contemporary forensic practice resonates strongly with our research objectives.

**The work presented here involved participants who are not professional interviewers. This might seem like a weakness of the study, as one might expect the AI's performance to be compared to that of law enforcement officers. However, it is often overlooked that most crimes against children are not discovered by professional investigators but, for example, by kindergarten or primary school teachers, social workers, neighbors, etc. The comparison group used by the authors in their study is therefore relevant because it addresses a social issue: is it better to use AI to listen to a child who is suspected of being a victim of abuse or violence, or should we allow the person who becomes aware of the abuse to interview the child, even if they have never been trained to perform this difficult task?**

Thank you for your suggestions. You are correct. According to Paula's (2011) interviews with 191 abused children, the findings showed that in children's first disclosures, 66.7% were made to parents, and disclosures to other family members accounted for 11.1%. This pattern of disclosure to non-professionals extends beyond family members: studies show that children frequently disclose to teachers (35%), community figures (33%), and school staff (30%), with considerably lower rates of disclosure to trained investigators (2.6%). This prevalence of disclosure to untrained individuals suggests both the potential utility of LLM interviewers and provides rationale for including naive participants as a comparison group.
In addition to the practical reasons mentioned above, another reason we chose to use an inexperienced group as the control group in this study is that the relationship between experience and the use of open-ended questions remains unclear. For example, Wolfman et al. (2016) found that investigators' experience in conducting investigative interviews with children did not predict their ability to use open-ended questions. Furthermore, Powell et al. (2014) found a negative correlation between experience in interviewing children and the ability to use open-ended questions. Therefore, we believe that comparing with an inexperienced group can effectively demonstrate the capabilities of large language models.
We now addressed these points both in the introductory section and in the discussion to make a stronger case for the basis of our choice of inexperienced groups.

“Studies have consistently demonstrated that children's initial disclosures of abuse often occur to non-professional individuals rather than trained forensic interviewers. In examining first disclosures, Magnusson (57) found that among 57 abused children aged 3-7 years, over 10% chose to initially disclose to their kindergarten teachers, significantly higher than first disclosures to police investigators and psychologists (2.6%). Similar patterns of initial disclosure were observed by Münzer et al. (58), with first disclosure rates of 10% to social workers and 5% to teachers. In terms of overall disclosure patterns, larger-scale studies have reinforced this trend. Lev-Wiesel et al. (59) reported that over 55% of 281 Israeli children disclosed to non-professionals including teachers, counselors, and social workers. Similarly, Postmus et al. (60), in a study of 708 abuse disclosure cases, found that 35% disclosed to teachers, 33% to community authority figures, 30% to school staff, and 30% to religious leaders. Among formal recipients, 33% disclosed to healthcare providers and 19% to counselors. This pattern of disclosure, particularly the prevalence of initial disclosures to non-professionals, suggests potential applications for LLM-based interviewing systems. Furthermore, it provides theoretical justification for including naive participants as a comparative baseline in examining LLM interviewer performance, as they represent a significant portion of real-world disclosure recipients.”

**Overall, the article is well-structured and organized. The abstract is clear and accurately reflects the content of the article. However, it might be helpful to include in parentheses what 'Recommended' and 'Not Recommended' questions are, as readers who are not specialists in this field (law enforcement officers, social workers, doctors, journalists, policymakers, etc.) may not immediately understand what these terms describe. A sentence could be: "Irrespective of the condition, recommended questions (XXX, XXX) elicited more correct information than not recommended ones (YYY, YYY, YYY, and YYY)."**

Response: Thank you for your advice. We have now included these examples in the sections of the article that frequently refer to question types to make the presentation easier to understand.

**The keywords are relevant. Perhaps a keyword related to 'questions,' which are the main measure of the article, should be added.**

Response: Thank you for your advice. We have now added a “Question Formulation” keyword to make it more comprehensive.

**Overall, the literature review, the research question, the description of the materials and procedure are well-written, relevant, and meet expectations. However, I am more cautious regarding the data analysis (results) and its description. The discussion is interesting and covers all the aspects addressed. The future directions and limitations are relevant and prompt the reader to reflect on what should be done in terms of experimental research and practical application in the field.**

Here are some comments and remarks.

____ INTRODUCTION ____

**P.4, L.78___ The description of international recommendations on the use of open-ended questions is insufficient. An uninformed reader who is not familiar with the topic will not understand what should be done and why. Examples of different types of questions are necessary, and these examples should be supported by data from the scientific literature. This literature can include studies on interview methods with witnesses and victims in general (see, for example, research on cognitive interviews, which contains many recommendations suitable for this manuscript), but especially those related to the NICHD. These elements are fundamental and should be addressed in a dedicated paragraph. A more detailed section on the NICHD and the necessity of its various phases should also be written, as it will help conceptualize future research and clarify the discussion.**

Response: Thanks to your suggestion, we have now included a more detailed description of the types of questions and NICHD in the introduction to elaborate on the various components of the investigative interviews with children recommended in NICHD, the implications of the different types of questions found in the study, and other information.

“The National Institute of Child Health and Human Development (NICHD) Investigative Interview Protocol represents one of the most extensively validated semi-structured approaches for conducting forensic interviews with children (8,22–24). The protocol progresses through distinct phases: introduction and ground rules, rapport building, practice narrative, substantive phase, and closure (21,25). Central to its effectiveness is the emphasis on open-ended prompts (See Table 1 for details) that elicit free-recall narratives from children, as these yield more accurate and detailed responses compared to focused or closed questions (22,26). “

| Question Category | Definition | Examples | Recommended Use | Effect on Child's Response |
| --- | --- | --- | --- | --- |
| Invitations/Open-ended prompts | Questions that allow free recall and give children control over disclosure | - "Tell me everything that happened"  - "Tell me more "  - "What happened next?" | Primary questioning strategy; should be used as much as possible | - Longest responses  - Most accurate information  - Most detailed accounts  - Child has control over narrative |
| Cued invitations | Open questions that follow up on details already mentioned by the child | - "You mentioned [detail]. Tell me more about that"  - "You said [event]. Tell me everything about that" | Should be used frequently to explore details mentioned by child | - Helps maintain focus while remaining open  - Elicits elaboration on specific topics  - Based on child's own words |
| Directive questions | Focused questions about previously mentioned information (who, what, when, where) | - "When did that happen?"  - "Where were you?"  - "Who was there?" | Use only when open-ended questions cannot elicit needed information | - More focused but still allow some elaboration  - Generally accurate |
| Option-posing/Forced choice | Questions providing specific options or requiring yes/no answers | - "Was it day or night?"  - "Did he hit you?"  - "Were your clothes on or off?" | Should be limited; use only when necessary details cannot be obtained through other means | - Very brief responses  - Risk of guessing - Lower accuracy  - May lead to acquiescence |
| Suggestive questions | Questions containing information not mentioned by child or implying desired response | - "He hit you, didn't he?"  - "That must have hurt"  - "Did he do other bad things?" | Should be avoided | - May lead to child providing false information during the interview and later on  - Can compromise testimony credibility  - Highest risk of inaccurate responses |

*Based on Lamb et al., 2018; La Rooy et al., 2015; Szojka & Lyon, 2024

**P.4, L.95 & L.100 ___ 'individual differences' --> this expression is unclear, and it is not clear whether it refers to the interviewees, the interviewers, or someone else. Not indicating this forces the reader to refer to the original article (which is not a major issue in itself, but it disrupts the flow of reading and, therefore, the strength of the authors' argument).**

Response: Thank you for your advice. Our reference to “individual differences” in this section focused on individual differences that led to inconsistencies between the group and individual effects of training. For example, a training program may be effective at the group level, but this effect will not be seen in each individual. We were not discussing in detail the specific individual traits that have an impact here. We have added a section here to describe in detail the impact of specific individual traits (e.g., personality traits) on training effectiveness.

“David and Lorraine (38) demonstrated that transfer of police training to field practice is influenced, both directly and indirectly, by multiple factors including trainee motivation, perceived training relevance and quality, and task preparedness. In the context of open-ended questioning, Melinder and Gilstrap (39) established a positive correlation between officers' self-regulatory capacity and their ability to employ open-ended questions effectively. Furthermore, research has explored the relationship between individual characteristics and overall investigative interviewing outcomes with children. Al Ali et al. (40) revealed significant associations between emotional intelligence and investigator performance. Personality factors, specifically openness, conscientiousness, and neuroticism, have also been identified as significant predictors of child interviewing effectiveness (15,41).”

**P.5, L.116 and following ___ This section is interesting, and the arguments presented are relevant and appropriate. Reading it made me think of the Self-Administered Interview (SAI), which does not require the intervention of a professional. It could be interesting to add a few sentences highlighting the strengths and weaknesses of this method compared to the use of AI and NICHD-type interviews (even though I am fully aware that the SAI has not been tested with children). The concept of Self-Report used in the SAI is also addressed later in this manuscript, P.6, L.151.**

Response: Thank you for the suggestion. The Self-Administered Interview (SAI) is a widely used self-report tool in witness investigations. While it allows witnesses to provide information without third-party interference and can elicit more accurate details (Lorraine et al., 2011), it also has limitations. For instance, using this tool requires individuals to have well-developed linguistic and cognitive abilities. However, younger children, whose language and cognitive abilities are still developing, might find it challenging to use such self-reporting methods effectively (Julie et al., 2012). Additionally, Del Castillo and Wright (2009) pointed out that children tend to disclose to people with whom they have close relationships, but self-report questionnaires cannot meet this relational requirement. We now added this to the introduction part.

“The Self-Administered Interview (SAI) is a widely used self-report tool for witness interviewing. While it allows witnesses to provide information without third-party interference and can elicit more accurate details (51), the self-reported methods also have limitations, especially with children. For instance, using this tool requires individuals to have well-developed linguistic and cognitive abilities. Therefore, younger children, whose language and cognitive abilities are still developing, might find it challenging to use such self-reporting methods effectively (52,53). Additionally, Del Castillo and Wright (54) pointed out that children tend to disclose to people with whom they have close relationships, but self-report questionnaires cannot meet this relational requirement. Therefore, it is important to have interviewers present appropriate questions to children.”

____ AIMS AND HYPOTHESES ____

**I generally agree with the three hypotheses put forward by the authors, but they should be justified by referencing key findings from the literature that support their claims. It is essential that the reader does not have to search for information and reconstruct the authors' argument and reasoning for their hypotheses.**

Response: Thank you for your advice. We have now added some detail to the hypotheses section by restating the rationale for each hypothesis before it is formulated, and distinguishing between exploratory hypotheses (hypotheses 1 and 2) and validating hypotheses (hypothesis 3)

“Based on the expectation that LLMs will better adhere to the interviewing guidelines (75,76) while naive human interviewers may not be able to do so (27–30), we formulated the following exploratory hypotheses:
Hypothesis1: Question Formulation: We expected the LLM (vs. naive human interviewers) to formulate (a) more recommended questions (e.g. directive questions, “Tell me what happened.”), both in absolute numbers and proportionally, and (b) less non-recommended questions (e.g. close-ended questions, “Did he touch the girl?”).
Hypothesis 2: Information Elicitation: We expected the LLM (vs. naive human interviewers) to elicit (a) more correct information and (b) less incorrect information from the children.
Based on previous research indicating that recommended questions can trigger children's free recall, whereas not-recommended questions may lead to false memories due to their suggestive nature (22,26), the following hypothesis was also tested:
Hypothesis 3: Irrespective of the interviewer type, we expected recommended (e.g. tell me what happened.) questions to elicit (a) more new correct information and (b) less incorrect information from the children compared to not recommended questions (e.g. did he touch the girl?)”

**Without any theoretical justifications, I could formulate opposing hypotheses based on a different line of reasoning: if I understand the functioning of LLMs correctly (I am not an expert in this field), these algorithms 'learn' a task or function by processing large amounts of data. In this case, CHAT GPT was trained on interviews of standard quality. In other words, the training data likely did not include best practices. For example, the training interviews probably contained many more 'not recommended' questions than 'recommended' ones. Therefore, how could CHAT GPT produce 'recommended' questions if it rarely encountered them during training? Based on this alternative (and perhaps completely incorrect and irrelevant) reasoning, we could expect, at best, 'equality' between humans and the machine (which is, of course, an invalid hypothesis), or at worst, a lower quality of questions posed by the machine compared to those posed by humans.**

Response: Thank you for your question regarding potential biases in LLM training data. Indeed, previous studies have documented various forms of bias in large language models, such as GPT-3.5's implicit racial bias in educational contexts, which likely stems from biased labels in training data (Melissa et al., 2024). While it's important to distinguish between implicit and explicit tasks. The documented biases typically emerge in implicit scenarios where the model's behavior is subtly influenced by patterns in its training data. However, our research focuses on explicit interviewing behaviors with clear, well-defined characteristics. The task of generating open-ended questions is fundamentally different - it relies on the model's ability to understand and follow explicit instructions rather than being influenced by implicit patterns. Through Instruction Tuning (Zhang et al., 2024), the model learns to comprehend the nature of "open-ended questions," and using Few-shot Learning (Gao et al., 2021), it can precisely generate such questions by drawing from its broad knowledge base while adhering to specific guidelines. This explains why the model can effectively generate recommended interview questions even if such examples might be underrepresented in its training data.

“Given that LLMs are the result of pre-training on vast amounts of data, it may prove challenging for them to extract relevant information from their training data and respond effectively. However, Instruction Tuning and Few-shot Learning techniques effectively improved this challenge. Through Instruction Tuning (67), the model learns to follow specific guidelines and principles, allowing it to focus on relevant knowledge rather than being overwhelmed by its broad training data. When combined with Few-shot Learning (68), the model can quickly adapt its vast knowledge to the specific requirements. Specifically, the example enables the model to extract interviewing patterns and behavioral protocols from demonstration cases (69), while Task Clarification establishes operational parameters and performance criteria for the interview process (70,71). This combination enables the model to generate appropriate questions that align with professional standards, effectively bridging the gap between its broad training and specific task requirements.”

____ METHOD ____

No comment


____ PARTICIPANTS ____

**The authors conducted and presented an a priori power calculation, which is relevant and appreciated. However, the effect size proposed (and assumed) by the authors (i.e., .500) seems to come out of nowhere. Without any justification, this gives the informed reader the impression that this calculation was included in the manuscript to justify a sample size constrained by reality (i.e., the authors only had access to the mentioned sample size and chose the effect size that would justify their imposed sample)**

Response: Thank you for your advice. Referring to Benia et al (2015) meta-analytic study of investigative interviews with children, we followed the effect sizes set by Hedges (1982), i.e., ES ≤ 0.2 is a small effect size, 0.5 is medium, and 0.8 is a large effect size. We chose a medium effect size here. We now explain the reasons for choosing this effect size in the manuscript.

“Hedges (77) suggests that an effect size ≤ 0.2 can be considered small, 0.5 medium and 0.8 a large effect, and this criterion could also be employed in investigative interviews with children (23). Based on this, a power-analysis with G*POWER showed that, assuming an effect size of .50 with α = .05, the sample size would result in a power of .706.”

___ MATERIALS ____

No Comment


____ PROCEDURE ____

My comments on the procedure mainly concern the instructions (prompts) given to the LLM.

**For example, these instructions do not mention the main NICHD recommendations, even though the coding variables used and presented on the following page are drawn from a meta-review on the NICHD. For instance, it is essential that the interviewee be allowed to give a 'free recall' following a very general prompt inviting the child to recall everything they remember. And the child should not be interrupted. These few guidelines are fundamental and distinguish a good interview from a bad one. I mention this so that the authors justify the absence of such instructions. In fact, readers who are well-versed in this specific topic will immediately notice the absence of mentions on these points. However, I know and clearly understood from reading the procedure that the children were never interrupted while speaking, whether by the human or the machine.**

Response: Thank you for your advice. As you pointed out, we did not explicitly specify in the Prompt the standard rules (e.g., NICHD) that human interviewers and LLMs should follow, because we wanted to compare the performance of inexperienced human interviewers with that of LLMs who had not been trained on a specific set of data. Therefore, the abstract nature of our prompts, as opposed to providing specific instructions, enables a more authentic assessment of the interviewing capabilities of both LLM and human interviewers in naturalistic settings. Nonetheless, we made it clear in the Prompt that open-ended questions should be used and that follow-up questions should be asked after the child has completed one response (i.e., the child should not be interrupted). However, as you point out, this is a shortcoming of this study, so we have also addressed this point in the Discussion section.

“Moreover, in the present study, although the prompts given to both human interviewers and LLMs employed advanced technology such as instruction tuning (67) and few-shot learning (68,70,71), it contained only abstract requirements rather than specific guidelines (such as NICHD protocols). As such, our findings solely reflect the baseline capabilities of LLMs. Although LLM performance is context-dependent, it remains an open question whether LLMs could effectively adhere to professional guidelines when provided with comprehensive professional instructions.”

**Table 2 mentions in its last paragraph that both the machine and the human should obtain a description of the adults present in the video. However, no data concerning the physical appearance of the individuals is presented in the manuscript. At most, we find mentions of certain 'dynamic' elements on P.14, L.309 and following, as well as in Table 5.**

Response: Thank you for your advice. In our data analysis, we did not separately analyze the appearance information of the adults in the videos, but labeled them as correct or incorrect information according to the coding rules. Static information about the adults included gender (male), attire (light-colored t-shirts and shorts, hats, and eyeglasses). We did not code information about the adult's looks because this information is ambiguous rather than objective. We have added a statement of these coding rules in this section.

“Static information about the adults included gender (male), attire (light-colored t-shirts and shorts, hats, and eyeglasses) and the same about the child present in the video. The appearance of both the adult and the child were not coded because this information was ambiguous rather than objective.”

____ CODING ____

**I believe that the choice of coding variables is one of the contentious points of this manuscript. For instance, I understand that correct, incorrect, and confabulations are sometimes redundant information (repeated multiple times by the same child) are counted each time as a "new" information. As a result, following this coding approach, a child who repeats 20 times that there was a man in the video will receive a score of 20 correct pieces of information, which is not consistent with the established literature. This coding method skews the results and, consequently, the interpretation of performance, which will appear far superior to what it actually is. A reported piece of information, whether correct or incorrect, should only be counted once. Saying 20 times that the adult was a man provides the same amount of information as saying 'it was a man' just once.**

Response: Thank you for your advice. In our study, we introduced “Unique Information” to distinguish between variables that were repeatedly mentioned by children and analyzed the absolute number and proportion of this variable. You can please refer to Tables 6,9 and to the results of Hypothesis 2, where we refer to the analysis of Unique Information.
“To separate between new and already mentioned information, a “Unique information” variable was introduced in the coding, which represented the total number of unique correct pieces of information mentioned by the child without repetitive counting of the same detail.”

**Moreover, the Accuracy Rate variable, which is frequently mentioned in the literature on judicial interviews, is missing. The accuracy rate should be calculated and statistically analyzed. It is calculated by dividing the number of UNIQUE correct pieces of information by the total number of UNIQUE pieces of information reported. This information is crucial and will help better interpret and understand the results of the analyses, which are sometimes contradictory or uninterpretable. I know I am asking a lot of the authors and that this will slow down the publication process (as I hope the editorial committee will ultimately decide to publish this manuscript), but the investment placed in obtaining this verbal data cannot be diminished due to an unstudied variable. Table 6 should therefore be modified to now include this new variable. Additionally, the authors can keep the 'cumulative' volumes of correct information, errors, and fabrications, but they must also report the volumes of UNIQUE information and, unfortunately, redo the analyses accordingly.**

Response: Thanks to your suggestion, we have now added a new variable called “Proportion of Unique Information” to refer to the proportion of unique details reported by each child out of the total number of Unique details in the video they watched. The MANOVA was also conducted on this data and found that there was no significant difference in this variable between Humans and LLMs. We have added the results in the Hypothesis 2 section, as well as a discussion towards this result.

“Moreover, regarding the proportion of unique details, which indicated the unique details reported by children as a percentage of total details in the video, results showed that no difference was found between the human participants (*M* = .10, *SD* = .06) and LLMs (*M* = .11, *SD* = .05), *F* (1, 76) = 1.05, *p* = .310.”

**Tables 4 and 5 could be moved to the appendices, as they provide little information relevant to understanding the authors' argument.**

Response: Thanks for the advice. We have removed both tables in the original text, but uploaded them in the supporting information of the supplementary submission.


____ CODING OF QUESTIONS AND ANSWERS ____

**Information about the coders is needed. How many were there? etc.**

Response: There were two coders conducting the coding. We now have added this in the coding part.


____ STATISTICAL ANALYSES ____

**It is not correct to perform multiple t-tests when dependent variables are most likely correlated with each other. Authors should use MANOVAs to analyze their data. All the most relevant indices should be presented in the text. Another interesting piece of information would be to report the correlations between the different variables in a correlation matrix. This information will make it easier to understand and interpret the sometimes contradictory results in this version of the manuscript.**

Response: Thank you for your advice. We have now performed the correlation analysis and presented the correlation matrix of the main variables in the descriptive statistics section. Secondly, as you have pointed out, because there are strong correlations between all the main variables, we have performed Manova analysis on the main data and revised the statistical analyses and results in the Manuscript. The results of the MANOVA were consistent with the original analysis.

“Given the strong correlations among our primary dependent variables (See Table 5 for details), a Multivariate Analysis of Variance (MANOVA) was conducted with Bonferroni correction. For dichotomous dependent variables (question authenticity), a non-parametric Mann-Whitney U test was employed.”

**I have noticed that the statistical values reported in the first two analyses (P. 21, L.379-384) are exactly the same (t, p, d, 95% CI). This may be a coincidence, but it's worth checking.**

Response: Thank you for your advice. Upon our inspection, the dependent variables analyzed in this section are inverse of each other correlated, i.e., a question is either a recommended question or a non-recommended question. Therefore, the analyses of these two variables are essentially the same, and therefore there is no problem with the results reported here. Also, given the stronger correlation between the dependent variables and the suggestions you mentioned, we have now reanalyzed these variables using MANOVA and revised the new results in the manuscript.

**The order of the dependent variables analyzed could be modified. Generally, we start with correct information, errors and affabulations, total and accuracy rate. Please note that this information is sometimes highly correlated, like correct information and accuracy rate. Care should be taken when presenting and discussing the results. The total volume of information and the accuracy rate are conceptually independent and should be the focus of the authors' discussion.**

Response: Thank you for your suggestion, we have now modified the presentation logic of the results section to present the results according to the order suggested by you. At the same time, we have added a description before presenting the results for unique information, explaining how this variable differs from correct information, in order to emphasize the importance of introducing it.

“In testing Hypothesis 2, the correct information reported by children was calculated cumulatively, whereby repeated instances of the same correct information were counted multiple times, potentially inflating the total volume of reported information. To address this limitation, "Unique Information" variables were introduced to capture the absolute quantity of non-redundant correct information provided by children. The variables were subsequently incorporated into the MANOVA analysis.”

**Certain terms, such as “marginally” (P. 22, L. 409), "significantly" (P.23, L. 436) and “partially” (P. 21, L. 384), should not be used in the presentation of results, as they could be misinterpreted by readers who are not specialists in statistics and its subtleties.**

Response: Thank you for your suggestion, we have now removed these words from the article to avoid unclear presentation of the results.

**Tables 1 and 7 could be placed in an appendix, as they do not contain information crucial to understanding the authors' arguments.**

Response: Thanks to your suggestion, we have now removed both tables from the manuscript and moved them to the supporting information, which has also been uploaded as supplementary material. Interested researchers can read this supporting information to understand the differences between typical AI and human interviews

**Decimals in numerical values should be two, with the exception of the p value.**

Response: Thank you for your advice. We have now changed all the data in the article to two decimal places

**Graphs showing total volumes of information and accuracy rates as a function of question type would be relevant and facilitate understanding of the phenomenon under study.**

Response: Thank you for your advice. We have included a set of figures in the Hypothesis 3 section showing the number and proportion of information obtained from recommended/non-recommended questions, and the number and proportion of information obtained for each question type, respectively.

**Table 10 could be structured around the two categories “recommended/Not recommended”. These should appear clearly in Table 10. It would also be relevant for a table like Table 10 to present all the means and standard deviations of the different variables according to both question type and origin (Human vs. LLM).**

Response: Thank you for your advice. We have now modified Table 10 (Now Table 6) to show the results more clearly. Also, we present the mean and standard deviation of the main dependent variable in Table 5 as part of the descriptive statistics.

____ DISCUSSION ____

**The discussion in its current form is interesting and takes up the results. Many of the comments are pertinent, particularly those concerning the study's limitations. The authors are genuinely self-critical, which will serve to improve their initial paradigm and thus, ultimately, the relevance of their work. However, I think it will have to be modified in the light of the results of the new analyses to be carried out.**

Response: Thank you for your suggestions for discussion. We've added the results of the proportion of unique information to the discussion, as well as the limitations of prompt, etc., to the discussion based on your other suggestions.

___ MINOR COMMENTS ___
**P.4, L.93 ___ mega-analysis ? ---> meta-analysis ?**

Response: Thank you for your advice. After our confirmation, the literature cited here should be “mega” and not “meta”. “Mega” analysis is an analysis based on combining the raw data from a number of studies, which is different from meta-analysis that combines the information based on group statistics.

Pompedda F, Zhang Y, Haginoya S, Santtila P. A Mega-Analysis of the Effects of Feedback on the Quality of Simulated Child Sexual Abuse Interviews with Avatars. Journal of Police and Criminal Psychology. 2022 Sep 1;37(3):485–98.

Reviewer #2:

**1. The objectives and hypotheses could be stated more clearly at the beginning of the manuscript.**

Response: Thank you for your suggestion, we have added a description to the first paragraph of the introduction to better present the hypothesized basis as well as the purpose of this study.

“Recent advances in Instruction Tuning and Few-Shot Learning technologies have enabled LLMs to demonstrate consistent and reliable performance across various professional domains, including medical diagnosis and legal investigation. Given our anticipation that LLMs would exhibit superior adherence to interview protocols, we hypothesized that LLMs would demonstrate enhanced performance compared to human interviewers in simulated child eyewitness interviews.”

**2. While a power analysis was conducted, it would be beneficial to provide more detail on how the sample size was determined and whether it was sufficient to detect meaningful differences.**

Response: Thank you for your suggestion. Our effect size selection was guided by Hedges' (1982) effect size interpretation criteria, from which we adopted a medium effect size. This choice is supported by Benia et al.'s (2015) meta-analysis of investigative interviews with children. The rationale for our effect size selection has been detailed in the Participants section. In the Data Analysis section, we report effect sizes for all significant differences, using Cohen's d for t-tests and η^2^ for MANOVA.

“Hedges (77) suggests that an effect size ≤ 0.2 can be considered small, 0.5 medium and 0.8 a large effect, and this criterion could also be employed in investigative interviews with children (23). Based on this, a power-analysis with G*POWER showed that, assuming an effect size of .50 with α = .05, the sample size would result in a power of .706.”

**3. More information on the randomization process for assigning children to either the LLM or human interviewer condition would strengthen the methodology section. Clarifying how randomization was implemented can help assess the validity of the results.**

Response: Thank you for raising this question. During recruitment, we first established all available experimental time slots, which both child and adult participants could independently schedule. Children and adults used separate registration forms, preventing them from seeing whether a time slot had already been booked by the other group. When a time slot was booked by both a child and an adult participant, the child would receive an interview from the human interviewer. If only a child had booked a time slot, they would receive an LLM-conducted interview with researcher assistance. If only an adult participant had booked a slot, that session would be canceled, and the adult participant would be asked to select an alternative time slot. All participants were instructed before scheduling not to book slots concurrent with children they knew. Through this registration procedure, the assignment of children to experimental conditions was entirely random.
We have now made this recruitment process clear in the participant information section.

“Participants scheduled sessions independently through separate registration systems. Children were assigned to either human-conducted interviews (when both child and adult participants booked the same slot, *n* = 38, *M_age_* = 6.97, *SD* = 0.82, including 21 (52.5%) male) or LLM-conducted interviews (when only a child booked the slot, *n* = 40, *M_age_* = 6.93, *SD* = 0.83, including 22 (57.9%) male), ensuring random assignment to experimental conditions.”

**4. The interaction between the LLM and the interviewer could be elaborated upon. For instance, detailing how the prompts were structured and the rationale behind the specific instructions given to both the LLM, and human interviewers would provide insights into the experimental design.**

Response: Thank you for your suggestion. We have provided a detailed explanation of the prompt components in the prompt section to demonstrate our rationale for prompt adjustments based on the latest developments in LLM prompting techniques.

“I want you to play the role of an interviewer. I am going to play the role of an 8-year-old child (There will be another child) [Role Playing, as suggested by instruction tuning (78)]. Your task is to ask me (the child) questions, to find out what happened in the video I (the child) just watched [Task Definition (70, 71), as suggested by prompt engineering].
Guidance: Abused children are often reluctant to talk about what they have experienced when they are interviewed by police officers due to shame and embarrassment. Also, if the interviewer uses leading questions, children are likely to give answers to such questions that contain inaccurate information [Example Passes (69), as suggested by few-shot learning]. I (The child) have viewed a video in which a child played a game with an adult. Your task as the interviewer is to figure out what happened in the video [Task Definition (70, 71), as suggested by prompt engineering]. Although there is no specific script for the interview, no questions should go beyond the purpose of finding out what happened in the game. Questioning about my (the child’s) personal information is NOT allowed [Constraint Setting (79, 80), and for data security].
The game might have involved conversation and photographs taken. Your task is also to get a description of the adult who participated in the game. Please try to always use open questions. I want you to do the interview by asking one question at each time and wait for my (the children’s) response [Providing specific instructions]. Then, you may keep asking until you feel comfortable about what happened [Stop Condition].”
**Text enclosed in square brackets explains the rationale for including the preceding sentence in the prompt. These bracketed explanations were not presented to either LLMs or human interviewers.**

**5. The delay in question posing by the LLM compared to human interviewers is an interesting finding. However, the implications of this delay on the flow of conversation and the children's responses should be discussed in more depth.**

Response: Thank you for your suggestion. We have restructured our discussion of the relationship between temporal delay and interview efficacy. To strengthen our conclusions, we have integrated theoretical frameworks from both Psychological Refractory Period and Cognitive Load Theory.

“In the present study, the responses of the child were transcribed and inserted into the LLM by the interviewer who then also read the next question provided by the LLM. Not surprisingly, this resulted in a difference in the delay between the child's response and the next question they were asked. This delay can be reduced in the future by automating the speech-to-LLM and LLM-to-speech processes. However, our findings revealed that despite longer question intervals in LLM-mediated interviews compared to human interviews, LLM interviews elicited more information from children. This phenomenon can be explained through several theoretical frameworks. According to the Psychological Refractory Period (PRP) theory (90), which posits that individuals experience a brief period of reduced ability to respond to a second stimulus immediately following the processing of an initial stimulus, this delay could have allowed children to complete their cognitive processing of the previous question and prepare for the next one (91–93). The Cognitive Load Theory (94,95) further suggests that these natural pauses might have reduced the overall cognitive burden on children's working memory, enabling more thorough information processing and retrieval (48,96,97). The slower pace of LLM interviews may have provided an optimal cognitive processing window for the participating children, supporting their retrieval processes. This is particularly beneficial for children who, compared to adults, typically require more time for information processing and response formulation. These findings suggest that the natural delay in LLM-mediated interviews might inadvertently create more developmentally appropriate interviewing conditions for children, ultimately leading to more effective information elicitation.”

**6. While t-tests and Mann-Whitney U tests were employed, a more detailed explanation of the statistical methods used, including assumptions and justifications for their use, would enhance the rigor of the analysis. Additionally, reporting effect sizes for all significant findings would provide a clearer understanding of the practical significance of the results.**

Response: Thank you for your suggestion. Following the identification of strong correlations among dependent variables, we reanalyzed our data using MANOVA. The manuscript has been updated with these new results, including effect sizes (η^2^ for MANOVA, Cohen's d for t-tests) for all significant findings.

**7. The distinction between incorrect and confabulated information is crucial. A more thorough explanation of how these categories were defined and coded would improve the clarity of the findings.**

Response: Thank you for your suggestion. To enhance the clarity of these three information categories and their distinctions, we have added the following exemplar in our methodological description:

“For example, consider a scene containing a single white cup on the table. Recall responses were categorized as follows: statements such as "there was a white cup on the table" were classified as correct information; statements like "there was a black cup on the table," where the object was correct but details were inaccurate (black versus the actual white), were classified as incorrect information; and statements such as "there was a white cup and a phone on the table" contained confabulated information, where mentioned objects (the phone) were not present in the original video scene.“

**8. The discussion could benefit from a more extensive exploration of how the findings relate to existing literature on child interviewing and the use of AI in investigative contexts. This would help situate the study within the broader field and highlight its contributions.**

Response: Thank you for your advice. We have cited more research on child abuse and children's investigative interviews in the discussion section and have discussed in more detail the direction of AI in legal investigations to make the results of the discussion section more comprehensive.

6. PLOS authors have the option to publish the peer review history of their article ([what does this mean?](https://journals.plos.org/plosone/s/editorial-and-peer-review-process#loc-peer-review-history)). If published, this will include your full peer review and any attached files.

If you choose “no”, your identity will remain anonymous but your review may still be made public.

**Do you want your identity to be public for this peer review?** For information about this choice, including consent withdrawal, please see our [Privacy Policy](https://www.plos.org/privacy-policy).

Reviewer #1: **Yes:** Samuel DEMARCHI

Reviewer #2: No
